# Associating mutations causing cystinuria with disease severity with the aim of providing precision medicine

**DOI:** 10.1186/s12864-017-3913-1

**Published:** 2017-08-11

**Authors:** Henry J. Martell, Kathie A. Wong, Juan F. Martin, Ziyan Kassam, Kay Thomas, Mark N. Wass

**Affiliations:** 1 School of Biosciences, University of Kent, Canterbury, Kent, CT2 7NJ UK; 2 Urology Centre, Guy’s and St. Thomas’ NHS Foundation Trust, London, SE1 9RT UK

**Keywords:** Cystinuria, Structural modelling, Computational predictions, Personalised medicine, ExAC

## Abstract

**Background:**

Cystinuria is an inherited disease that results in the formation of cystine stones in the kidney, which can have serious health complications. Two genes (SLC7A9 and SLC3A1) that form an amino acid transporter are known to be responsible for the disease. Variants that cause the disease disrupt amino acid transport across the cell membrane, leading to the build-up of relatively insoluble cystine, resulting in formation of stones. Assessing the effects of each mutation is critical in order to provide tailored treatment options for patients. We used various computational methods to assess the effects of cystinuria associated mutations, utilising information on protein function, evolutionary conservation and natural population variation of the two genes. We also analysed the ability of some methods to predict the phenotypes of individuals with cystinuria, based on their genotypes, and compared this to clinical data.

**Results:**

Using a literature search, we collated a set of 94 SLC3A1 and 58 SLC7A9 point mutations known to be associated with cystinuria. There are differences in sequence location, evolutionary conservation, allele frequency, and predicted effect on protein function between these mutations and other genetic variants of the same genes that occur in a large population. Structural analysis considered how these mutations might lead to cystinuria. For SLC7A9, many mutations swap hydrophobic amino acids for charged amino acids or vice versa, while others affect known functional sites. For SLC3A1, functional information is currently insufficient to make confident predictions but mutations often result in the loss of hydrogen bonds and largely appear to affect protein stability. Finally, we showed that computational predictions of mutation severity were significantly correlated with the disease phenotypes of patients from a clinical study, despite different methods disagreeing for some of their predictions.

**Conclusions:**

The results of this study are promising and highlight the areas of research which must now be pursued to better understand how mutations in SLC3A1 and SLC7A9 cause cystinuria. The application of our approach to a larger data set is essential, but we have shown that computational methods could play an important role in designing more effective personalised treatment options for patients with cystinuria.

**Electronic supplementary material:**

The online version of this article (doi:10.1186/s12864-017-3913-1) contains supplementary material, which is available to authorized users.

## Background

Cystinuria is an inherited disorder resulting in urinary dibasic aminoaciduria [1]. The clinical presentation is varied; ranging from some patients having stone episodes every few months to other patients having only one stone in their lifetime. It is primarily caused by mutations in two genes; SLC3A1 encodes the neutral and basic amino acid transport protein (rBAT) and SLC7A9 encodes the light chain b amino acid transporter b(0+)AT [2, 3]. These two proteins form a dimer linked by a disulphide bridge [4]. b(0+)AT contains 12 transmembrane helices that form the channel through which dibasic amino acids (cystine, lysine, arginine and ornithine) are transported into the cell with the exchange of neutral amino acids. rBAT has a single transmembrane domain and a large extracellular domain. There is evidence to suggest that the extracellular glycosidase domain has a role in cystine transport but not the other dibasic amino acids [5]. rBAT also requires chaperones to fold correctly and some mutations have been linked with incorrect folding of the protein and/or trafficking to the plasma membrane [6].

Experimental studies suggest that rBAT may function as an activator of b(0+)AT [3, 7] but the functional role of rBAT remains unclear, although it is required for efficient transport to occur. Mutations in either of these two genes can result in defective transport of dibasic amino acids across the renal tubular membrane and intestine [8, 9]. In the kidneys, this results in cystine accumulating in the urine and forming stones.

SLC3A1 mutations are inherited in an autosomal recessive pattern whilst mutations in SLC7A9 can be regarded as inherited in an autosomal dominant pattern with incomplete penetrance [10]. In SLC3A1, mutations in both alleles of the gene are required for disease presentation. In SLC7A9, some patients only have one mutation in one allele and can form cystine stones [11].

Many mutations have been identified in both SLC3A1 and SLC7A9 in individuals with cystinuria [12, 13]. Frame shift, deletion, duplication, splice site and nonsense mutations typically result in large effects on the encoded protein and therefore its protein structure or function. Most mutations described in Cystinuria however, are missense mutations resulting in the change of a single amino acid in the protein. The effect of a missense mutation can range from having no effect on protein function to rendering it non-function. For many of the missense mutations in SLC3A1 or SLC7A9, without further analysis it is not clear what effect they have on protein function and how they lead to disease presentation.

The sequencing of many people has demonstrated that each individual has between 4 and 5 million genetic variants compared to the reference human genome [14]. Some of these variants will cause disease or increase the risk of disease, however it is difficult from this large set of variants to identify those that are most likely to have a phenotypic effect and may have a role in disease. As a result many computational methods have been developed to predict if a genetic variant is likely to be deleterious (reviewed in [15]). These methods largely focus on the analysis of non-synonymous single nucleotide variants (nsSNVs) and use many different features from sequence conservation to structural and functional information. Methods include SIFT [16, 17], PolyPhen2 [18], SuSPect [19], VarMod [20], SNAP [21], Mutation Assesor [22], FATHMM [23], CADD [24] and Condel [25].

We recently proposed that protein structural modelling and analysis of mutations present in cystinuria could be used to further our understanding of how mutations alter the function of the transporter and the extent of functional effect caused by each mutation [26]. Here we perform an extensive literature survey of clinical studies to identify the range of different mutations associated with cystinuria. A structural analysis of all the identified single point mutations present in rBAT and b(0+)AT is performed to investigate the effect of the mutations on the transporter structure and function. We also compare these mutations that have been reported to cause cystinuria with the natural variation of SLC3A1 and SLC7A9 present in the large population study of genetic variation ExAC [27]. Finally, the ability of automated predictors to assess the effect of cystinuria associated mutations is considered using clinical data from a cohort of 74 patients [28].

## Methods

### Cystinuria literature survey

A literature search was performed to identify all clinical studies of cystinuria patients. PubMed was searched with the terms “cystinuria” “cystinuria mutation” and “SLC3A1” and “SLC7A9”. Papers were first filtered on the basis of being original articles or reviews, with reviews discarded. Further filtering was performed by reading the abstracts of all papers to check for relevance. Those that were assessed to be relevant were then read fully and any relevant data extracted.

### ExAC data

Genetic variation data was downloaded from the Exome Aggregation Consortium (ExAC) browser, for both SLC3A1 and SLC7A9, on 28/10/2016 [27]. This data set was then filtered to contain only variants that affect canonical transcripts, and then further filtered to contain only non-synonymous single nucleotide variants (nsSNVs). This resulted in a set of 318 and 144 nsSNVs not known to be associated with cystinuria for SLC3A1 and SLC7A9, respectively.

The ExAC data was used to determine the allele frequencies of the variants identified by the literature search to have a role in cystinuria. In addition, all variants present in ExAC that were not identified to have a role in cystinuria form the SLC3A1 and SLC7A9 ExAC only variant sets.

### Structural modelling and analysis

The protein structures of rBAT and b(0+)AT were modelled using the Phyre2 web server [29]. Functional sites of the protein were modelled using multiple methods. The ligand binding sites including the amino acid, sugar and calcium binding sites were modelled using 3DLigandSite [30, 31] and *firestar* [32]. Protein stability predictions were made using mCSM [33] for all nsSNVs.

Residue conservation in rBAT and b(0+)AT was calculated using the following approach. Homologues were identified using BLAST [34] to search the UniProtKB with default parameters [35]. A multiple sequence alignment and a phylogenetic tree were generated for each of these sets of homologues using Clustal Omega with default parameters [36, 37]. For each gene, the multiple sequence alignment, phylogenetic tree, and phyre2 structural model were submitted to ConSurf [38], which was run using the Bayesian prediction method with all other parameters set to default. The proteins used for the alignment, and the species that they come from, are shown in Additional file 1: Tables S1 and S2. For the 2 proteins, there were 158 common species, 87 species unique to the alignment of rBAT, and 45 species unique to the b(0+)AT alignment. This shows that the majority of the species used in the two alignments are the same, and there is not a large difference in the species distributions of the two alignments. This means that reasonable comparisons of conservation between the two proteins can be made.

### Clinical data

Phenotypic data associated with cystinuria was available for a cohort of 74 patients in the UK [28], consisting of 41 patients with mutations in SLC3A1, 32 in SLC7A9 and one patient without a mutation in either SLC3A1 or SLC7A9. Available phenotypic data included, urinary dibasic amino acids levels for cysteine, ornithine, arginine and lysine, the age of disease presentation, and the number of stone episodes and number of interventions over a three-year period. Two of the patients in this cohort were removed from this study as they had mutations in both SLC3A1 and SLC7A9.

### Automated prediction of the effect of mutations

The mutations found in the literature search were submitted to SIFT [17], PolyPhen2 [18] (using both predictive models HumDiv and HumVar), MutationAssessor [22], FATHMM [23], Condel [25] and CADD [24] using default settings. Condel and CADD differ from the other prediction methods in that they integrate predictions from individual methods to create an overall prediction. For example, Condel uses predictions from PolyPhen2, SIFT, Mutation Assessor, and FATHMM to make predictions.

The different methods make predictions in different categories, SIFT only predicts two categories “Tolerated” and “Damaging”, as do FATHMM, CADD, and Condel (“neutral” and “damaging”), while PolyPhen2 categorises mutations into three categories “benign”, “possibly damaging” and “probably damaging” and MutationAssessor predicts four categories (“neutral”, “low”, “medium”, and “high”).

PolyPhen-2 is available using two different training models, HumDiv and HumVar. These two models agree for 47 of 58 mutations in b(0+)AT and 81 of 94 mutations in rBAT. As we want to distinguish between mildly deleterious and more severe mutations, the remaining analyses consider only the results using the HumVar training model. However, as shown above the two models give similar results.

### Grouping patients by mutation severity and comparison of phenotypes

The different categories of the prediction methods make analysis of the overlap of agreement between the methods difficult to assess. Therefore, the prediction scores made by each of the automated methods were classified into two groups, either mild or severe and these two groups were then associated with a score mild = 1 and severe = 2. Multiple thresholds for grouping mutations were tested for each of the prediction methods (see Additional file 1: Tables S3 and S4), starting from the recommended threshold for separating deleterious and neutral mutations for the specific method (see Table 1). The stringency of the threshold was then increased incrementally to separate the high confidence predictions from the medium confidence predictions. Thresholding above the cut-offs for deleterious vs neutral variants was necessary because these methods are designed to predict even mildly deleterious variants as deleterious, and we want to separate mild from severe mutations.

**Table 1:** Mutation severity prediction score thresholds used for each method, based on stabilisation of group numbers above the recommended deleterious threshold

Method	Standard Deleterious Threshold	Threshold for Mutation Severity Score of 1	Threshold for Mutation Severity Score of 2
SIFT	Score < 0.05	Score > 0.025	Score ≤ 0.025
PolyPhen2	Score ≥ 0.5	Score < 0.80	Score ≥ 0.80
Mutation Assessor	Score > 1.9	Score < 2.7	Score ≥ 2.7
FATHMM	Score ≤ −1.5	Score ≥ −8.5	Score < −8.5
Condel	Score > 0.522	Score ≤ 0.672	Score > 0.672
CADD	Score ≥ 15	Score < 27.5	Score ≥ 27.5

Frameshift, deletion, splice site and nonsense mutations are typically likely to have a significant effect on protein function and were therefore all assigned scores of 2. This may represent a simple scoring scheme but given the different categories and scoring scales of the different methods it appeared to be the most appropriate.

Using the mutation scores from the predictive methods, each patient was assigned an overall severity score for the mutations that they have. As SLC7A9 mutations show dominant inheritance with incomplete penetrance, for patients with SLC7A9 mutations, patient scores were the total of the scores for the individual mutations in each allele. This results in scores ranging from one (only one mild mutation present) to four (patient has two mutations classified as severe, one in each allele).

A similar approach was taken for SLC3A1, but inheritance of cystinuria from SLC3A1 mutations is autosomal recessive, therefore mutations are required in both alleles to have the disease. For each patient, it was considered that the allele with the worst mutation would not be expressed while the other allele would be. For example, an individual with two mutations scored at 1, would have an overall score of 1, as would a patient with one mutation scored at 1 and the other at 2. Finally, an individual with two severe mutations would score 2 overall. This strategy is valid for our dataset, because no individual has more than one mutation in a single allele of SLC3A1.

The properties of each set of data were compared using the Wilcoxon rank sum test to find any statistically significant differences between the groups. All *p*-values were corrected for multiple testing using the Bonferroni method.

Statistical figures were produced using the R statistical package, version 3.2.1 [39]. Additionally, plots with axes gaps were produced using the R package ‘plotrix’ [40].

## Results

A literature search identified 52 articles, consisting of 49 original articles and three reviews. All of the original articles were deemed relevant from the abstract and read in full to extract data. From the clinical studies we identified a total of 94 SLC3A1 and 58 SLC7A9 cystinuria associated point mutations.

The 94 unique nsSNVs in SLC3A1 affect 81 different amino acid positions as 10 residues have two variant amino acids and residue p.Arg365 has four different variant amino acids present. For SLC7A9 the 58 nsSNVs affect 55 different amino acid positions with only residues 105, 195 and 333 having two different variant amino acids.

### Initial comparison of Cystinuria associated mutations with variation present in a large population.

The ExAC resource [27] provides access to the variant frequencies from over 60,000 individuals. We identified all variants present in SLC7A9 and SLC3A1 (Additional file 1: Tables S5 and S6) to investigate the variation present in a large population of individuals and compare variants/mutations associated with cystinuria and those not associated with the disease. Worldwide prevalence of cystinuria is estimated at 1 in 7000 [41], though variation by geographical location is large (1 in 100,000 in Sweden [42], and 1 in 2500 in Libyan Jews [12]. Using the wordlwide prevalence, in the ExAC set of just over 60,000 individuals, we would therefore expect approximately nine individuals to have the disease.

**Fig. 1:**
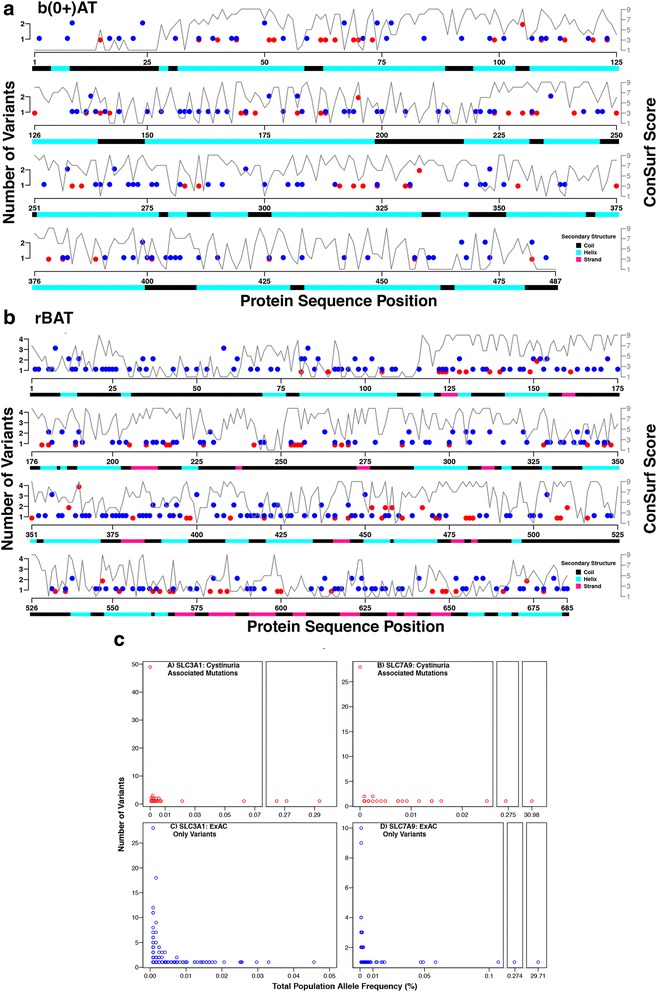
Mutations present in b(0+)AT (SLC7A9) and rBAT (SLC3A1) in patients with cystinuria. Plots of the sequence of (**a**) b(0+)AT and (**b**) rBAT. For each protein the location of cystinuria associated mutations is shown (*red circles*) with the position of variants present in ExAC (*blue circles*). The conservation score is shown (*grey line* with values ranging from 1 to 9). The *lower bar* shows the protein secondary structure. **c** Total population allele frequencies based on the ExAC data set. Each point represents an allele frequency. Multiple variants may have the same allele frequency, and the number of variants with the specific allele frequency is represented by the position of the point on the Y axis. The individual plots correspond to the four different sets of variants. C(A.) Variants of SLC3A1 reported to be associated with cystinuria. C(B.) Variants of SLC7A9 reported to be associated with cystinuria. C(C.) Variants of SLC3A1 not reported to be associated with cystinuria but present in ExAC. C(D.) Variants of SLC7A9 not reported to be associated with cystinuria but present in ExAC

The vast majority of cystinuria associated variants in both SLC7A9 and SLC3A1 occur very rarely with an allele frequency of less than 0.01% and many are not present in the ExAC dataset (allele frequency of 0% - Fig. 1c). This suggests that these variants are under purifying selection and that these mutations are deleterious. A few disease-associated variants have much higher frequencies (between 0.27%–31%). The cystinuria associated variant p.Val142Ala in SLC7A9 has an allele frequency of 31% indicating that it regularly occurs in individuals. Given the high frequency of this variant it is likely that it has a limited effect on SLC7A9 function as cystinuria is a rare disease, this is reinforced by the low evolutionary conservation of residue 142 in the protein (ConSurf score of 1).

Many non-disease associated variants in SLC7A9 and SLC3A1 also have low frequency (<0.01%; Fig. 1c). For SLC7A9 there are a few variants with higher frequencies, and a considerable number more for SLC3A1. This demonstrates that there is limited variation in SLC3A1 and SLC7A9 in the population. The higher frequency of variants in SLC3A1 may reflect that it has autosomal recessive inheritance, whereas SLC7A9 inheritance is autosomal dominant with incomplete penetrance. Thus, a single SLC7A9 allele can result in cystinuria.

At the protein level there appears to be some clustering of the rBAT cystinuria associated point mutations in sequence (Fig. 1b). For example, there are some mutated positions that occur in stretches throughout the protein (including 121–124, 253–256, 480–482, 552–568). While some ExAC variation occurs in the same sequence regions, there appears to be less clustering of these variants and ExAC only variants largely occur in parts of the protein sequence where cystinuria associated mutations are not present (Fig. 1b).

For b(0+)AT, unlike rBAT, there is less evidence of clustering of the amino acids that are mutated, with no runs of residues being mutated and only a few examples of adjacent residues being mutated (Fig. 1a). Again, there is limited overlap with ExAC variation data (Fig. 1a).

**Fig. 2:**
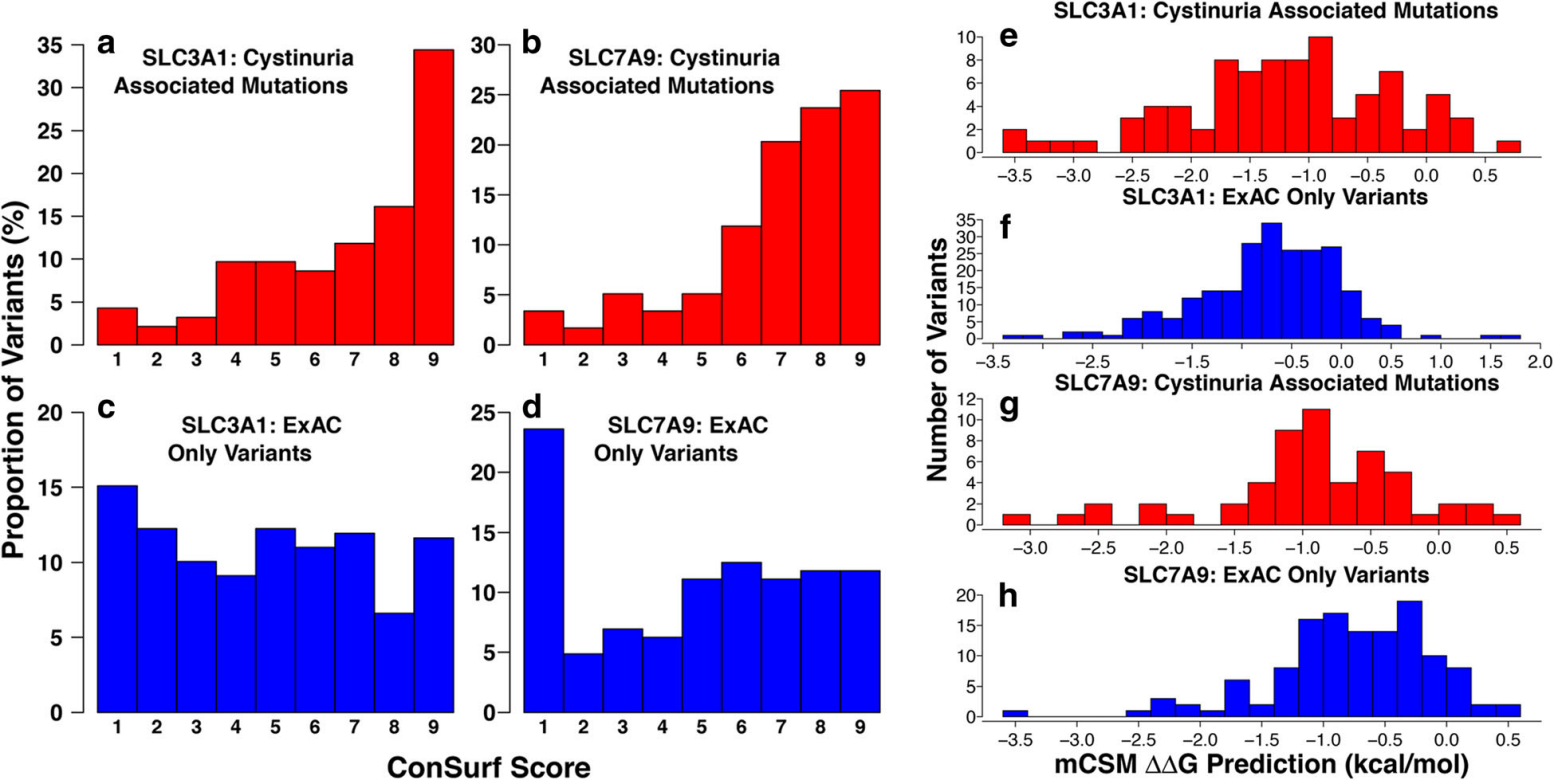
Conservation of nsSNVs in SLC7A9 and SLC3A1 and their predicted effect on protein stability. **a**-**d** Distribution of Consurf conservation scores for nsSNVs in SLC7A9 and SLC3A1 that are either i) present in individuals with cystinuria (**a** and **b**) or ii) present in the ExAC dataset (**c** and **d**). ConSurf scores vary between 1 and 9, with 9 being highly conserved and 1 being not conserved. **e**-**h**). Effect of nsSNV on protein stability predicted by mCSM. mCSM predicts the change in Gibbs free energy (kcal/mol) negative values indicate destabilisation and positive values stabilisation

The conservation of each variant position was calculated using ConSurf (see Methods). The conservation scores range from 1 to 9, with 1 being the most variable and 9 the most conserved. For both genes, there is a clear difference in the distributions between the mutations known to be associated with cystinuria and the variants only found in ExAC (*p* = 1.69e-10 for SLC3A1, and *p* = 2.078e-06 for SLC7A9, Wilcoxon rank sum test) (Fig. 2). The cystinuria associated mutations of both genes are predominantly at positions with high ConSurf scores, suggesting that these positions are of high importance to the function of the protein. This skew is larger for SLC7A9, where ~80% of the mutations have ConSurf scores between 6 and 9 (Figs. 1a-b and 2a-b). Conversely, for both genes, a large number of variants that are not known to be associated with cystinuria have ConSurf scores of 1 (Figs. 1a-b and 2c-d), suggesting that the functional roles of these positions are minimal. This agrees with these variants being neutral, as such positions are less likely to have an effect on protein structure or function. Around 40% of the positions of ExAC only variants have ConSurf scores between 6 and 9, it is possible that they may have some effect upon protein function or that the variants observed conserve the property of the wild type amino acid more so than the cystinuria associated mutations. We did not observe a correlation between ExAC allele frequency and ConSurf conservation score (Additional file 2: Fig. S1).

### Protein structural modelling

**Fig. 3:**
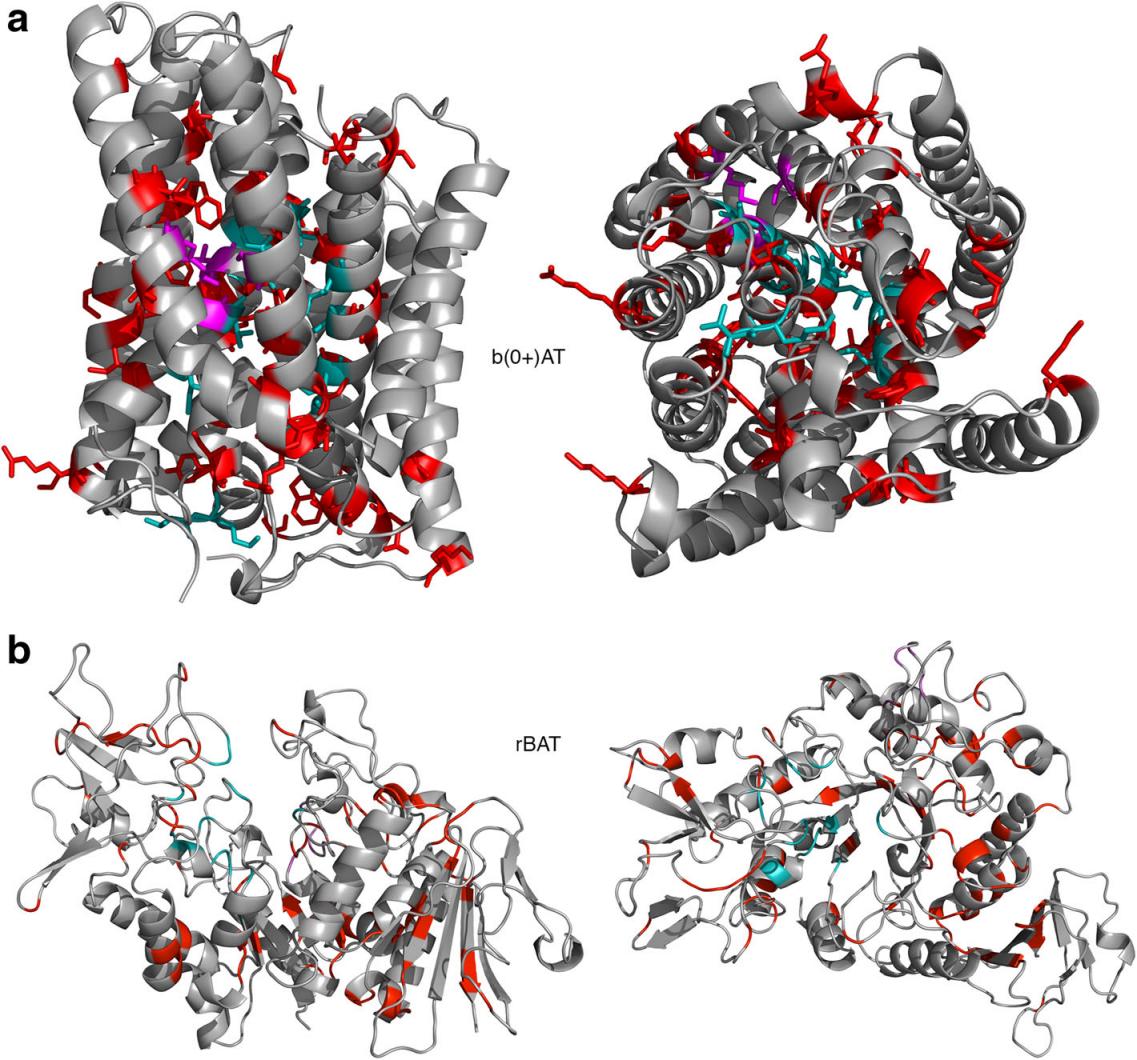
Structural models of rBAT and b(0+)AT. For both proteins cystinuria associated mutations are coloured *red*. **a** Model of b(0+)AT. Residues modelled to contact the transported amino acids are coloured *cyan*. The conserved p.Lys184 and residue coordinating with it are coloured *magenta*. **b** Model of rBAT. The modelled sugar binding site residues are coloured *cyan* and the predicted calcium binding site is *magenta*

To investigate where in the protein structure the cystinuria associated variants occur and to analyse the effect they may have on protein structure and function, protein structural modelling of the protein was performed. Phyre2 [29] generated high confidence structural models of both b(0+)AT and rBAT (Fig. 3). For rBAT, the extracellular alpha amaylase-like domain was modelled using the structure of Bacillus Cereus oligo-1,6-glucosidase [43] as a template (pdb code: 1uok). The structure of a glutamate and γ-aminobutyric acid antiporter [44] (pdb code: 4DJI) was used as the template structure for modelling b(0+)AT.

The b(0+)AT protein transports dibasic amino acids into the cell in exchange for neutral amino acids. There are therefore two sites for amino acid binding, one on each side of the transporter. The outward facing binding site was modelled using 3DLigandSite and firestar using the Arginine bound to another related APC transport (AdiC, pdb code: 3OBM) [43]. To model the inward facing conformation, the putative binding site identified for ApcT (another member of the APC transporter family) was mapped onto our model [45] (Fig. 3a).

Studies have proposed that Lys158 in ApcT has a role equivalent to sodium in sodium dependent transporters [46]. This lysine is conserved in b(0+)AT (Lys184) and three of the four residues coordinating with it are also conserved (Gly41, Ile44, Ser312).

Overall potential functional residues identified in b(0+)AT for amino acid binding were: Ile38, Thr42, Ile43, Ser46, Gly47, Val50, Thr91, Lys92, Leu117, Lys121, Ser124, Ile128, Trp230, Ala231, Tyr232, Ile371. As the mechanism of transport is not clearly understood there are likely to be further residues that are functionally important that have not been identified here.

In rBAT the residues predicted to have a potential functional role in the alpha amylase domain for sugar binding were: Asp172, Tyr175, His215, Val258, Tyr259, Phe278, Met279, Gln282, Ser312, Asp314, Ala315, Phe318, Glu384, Asp449 (Fig. 3b). Additionally, Asp133, Asn135, Asp137, Asn139, Asp141 are predicted to bind calcium (Fig. 3b). However, it is not clear if rBAT binds sugar molecules or if it has an alpha amylase enzyme activity.

### Structural analysis of mutations in b(0+)AT

Initial analysis of the substitutions that occur in b(0+)AT shows that for only 22 of the 58 point mutations the type of amino acid is not changed (Table 2). The majority of residues that are mutated are hydrophobic and for more than half of the changes (25 of 45) the mutated residue is polar or charged. This shows that mutations are regularly introducing charge into the protein.

**Table 2:** Type of amino acid change for mutations in b(0+)AT

	Mutation Amino Acid Type					
Original Amino Acid Type		Hydrophobic	Polar	Positive	Negative	Total
	Hydrophobic	20	10	12	3	45
	Polar	5	0	1	1	7
	Positive	2	1	1	1	5
	Negative	0	0	0	1	1

The likely effects of the mutations fall into a few categories (full analysis details in Additional file 1: Table S7). Firstly, some mutations alter residues with a functional role (e.g. ligand binding) or they are located close to functional sites. Secondly, some mutations seem likely to alter protein conformation as they either introduce charge or change the size/shape of the sidechain (often in buried or densely packed regions of the protein). Finally, some mutations are located on the protein surface and they could affect the interaction with the membrane or with rBAT.

**Fig. 4:**
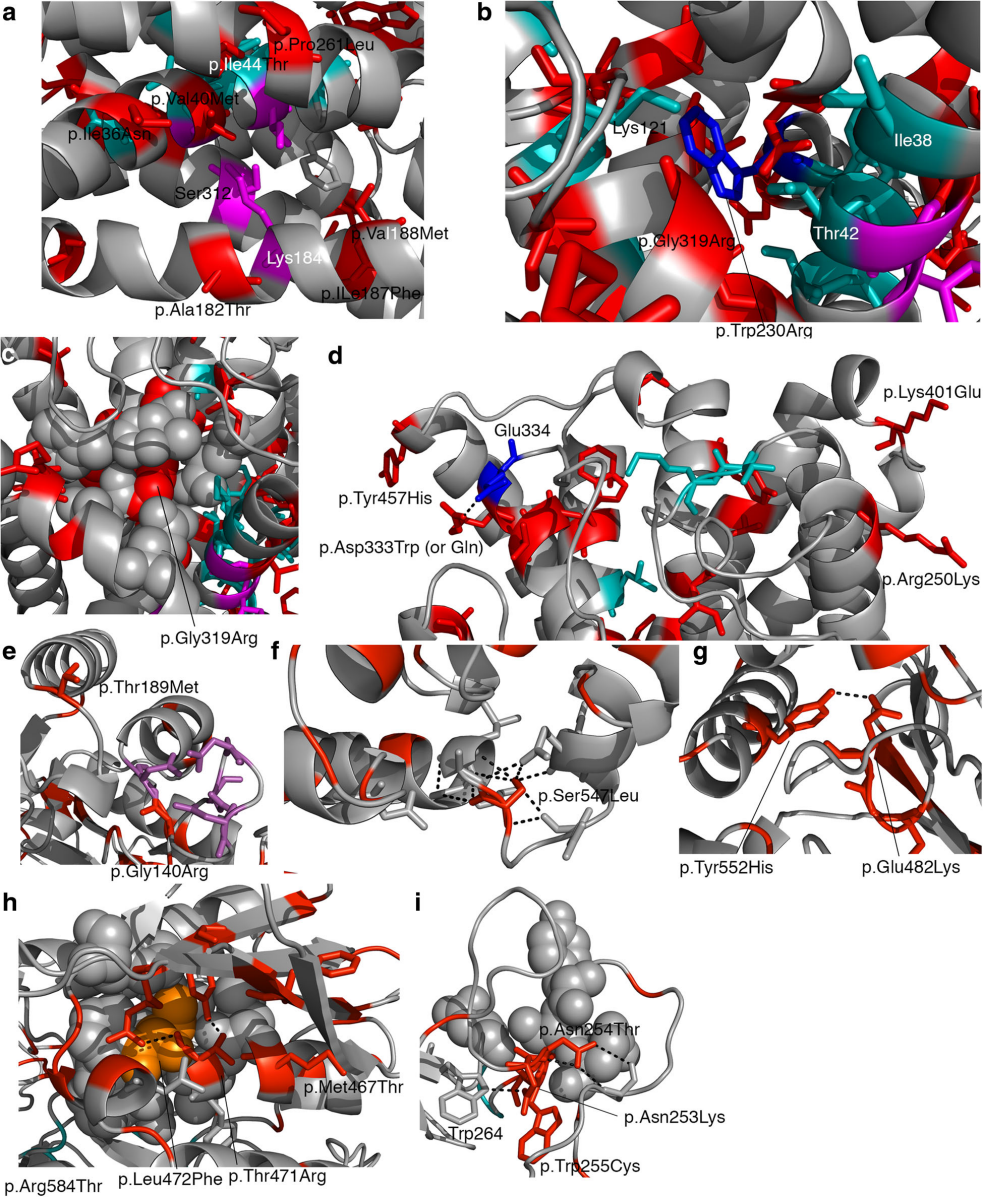
Mutations in b(0+)AT and rBAT. In all images the mutated residues are displayed as *red sticks* in their wild type format. Predicted functional residues are *coloured cyan*. Hydrogen bonds are shown as *dashed black lines*. Images **a**-**d** refer to b(0+)AT and images E-I refer to rBAT. **a** p.Ile187Phe and p.Ala182Thr mutations are adjacent to p.Lys184 which is thought to play a role equivalent to sodium in sodium dependent transporters. **b** p.Trp230Arg (*coloured blue*) is adjacent to multiple functional residues. **c** The mutation p.Gly319Arg occurs in a buried region (p.Gly319 shown in red spheres). **d**. Mutations close to the end of transmembrane helices may reduce stability in the membrane. **e** mutations occurring close to the predicted calcium binding site in rBAT. **f** mutation p.Ser547Leu will remove hydrogen bonding. **g** p.Tyr552His and p.Glu482Lys as wild type form a hydrogen bond, it is not clear if this will be retained upon mutation. **h** multiple mutations present in a single region. p.Leu472Phe (*orange spheres*) will result in increased size in well packed area. Other mutations will remove hydrogen bonding. I) Mutations occur in residues 253–256

There are a set of mutations close to the functional residue Lys184, which is likely to function in an equivalent way to sodium in sodium dependent transporters. One of the residues thought to coordinate with Lys184, Ile44, is mutated to Thr (Fig. 4a). Additionally, in the same area there are the mutations p.Ile36Asn, p.Val40Met, p.Ala182Thr, p.Ile187Phe, p.Val188Met and p.Pro261Leu (Fig. 4a). Many of these mutations seem fairly conservative, and suggest that minor changes to the conformation of the protein, through altered packing of sidechains may be sufficient to alter function. This may be particularly relevant as helix 1 (containing p.Ile36Asn, p.Val40Met, p.Ile44Thr) is thought to undergo conformational change during transport and the other side of the helix contains multiple residues that are likely to have a role in binding the transported amino acids (Fig. 4a – cyan coloured residues).

Other mutations are close to the residues likely to have a functional role in transporting the amino acids. One of these functional residues, Trp230, is mutated to Arg (Fig. 4b) and there are multiple other mutations in the same area that are close to functional residues (Fig. 4b-d).

b(0+)AT contains many hydrophobic amino acids, which are often tightly packed. In multiple examples, a smaller hydrophobic is replaced by either a polar/charged amino acid or a larger hydrophobic (for example p.Gly319Arg - Fig. 4c).

A final group of mutations may affect the interactions of the protein with the lipid bilayer and its stability. Most of these mutations either introduce (p. Tyr99His, p.Ala109Thr, p.Cys137Arg, p.Phe140Ser, p.Gly195Arg, p.Tyr457His), remove (p.Arg171Trp, p.Asp333Trp) or alter charge (p.Arg250Lys, p.lys401Arg) mainly at the end of helices on the protein surface near the end of the membrane (examples shown in Fig. 4d). Another possible impact of mutations on the protein surface of b(0+)AT (and also rBAT) is that they interfere with the dimerization of the two proteins. However, little is known about how these two proteins interact and what residues are involved in the interaction, so predictions of the impact on dimerization were not possible.

### Structural analysis of mutations in rBAT

While rBAT has an alpha-amylase like extracellular domain, the functional role of this domain has not been well established. Overall there are few mutations present (only three) in or near the predicted functional residues (based on possible sugar and calcium binding sites) (Fig. 3b). This suggests that these residues may not be functional in rBAT, otherwise mutations would be expected to occur here as was seen for mutations in b(0+)AT (although ConSurf shows that these residues are highly conserved, 11 of the 14 have scores of 8 or 9). It suggests that we do not know what residues are functionally important in rBAT and what function they perform. This makes the structural analysis difficult.

Despite this, a few mutations are located close to “functional” regions of the protein. p.Arg137Gly is one of the residues predicted to bind Calcium, mutation to glycine would lose the positive charge in this region. Similarly, p.Gly140Arg is present within the loop where calcium is modelled to be bound (Fig. 4e). This position is completely invariant in homologues (with a maximum ConSurf score of 9) suggesting an important structural/functional role for this residue. Introduction of a positively charged arginine may be expected to interfere with the binding of the positively charged calcium ion (assuming that Calcium does bind here). p.Thr189Met is located in the alpha helix adjacent to the calcium binding site so it is possible that destabilisation here could affect the calcium binding site (Fig. 4e). Three of the mutations (p.Met381Thr, p.Tyr397Cys, p.Gly398Arg) are close to what would be the active site if the protein was an active hydrolase. p.Met381 is highly conserved in orthologues and the mutation to threonine could introduce a polar contact with p.Asp369. Similarly p.Gly398Arg would introduce a charge and a larger sidechain. For p.Tyr397Cys the mutation is likely to remove a hydrogen bond (see below).

Overall the structural analysis suggests that, for the majority of the rBAT mutations observed, they may have an effect on the structure or stability of the protein (full structural analysis details in Additional file 1: Table S8). These mutations fall into two main groups. In the first group, a hydrophobic amino acid is replaced by a polar or charged amino acid (examples are p.Tyr151Cys, p.Leu205Ser, p.Leu300Ser, p.Tyr397Cys, p.Tyr461His, p.Met467Thr, p.Ile445Thr, p.Tyr579Asp, p.Phe599Ser) where the hydrophobic side chain is typically buried and packed against other hydrophobic side chains. Of the 49 hydrophobic sidechains that are mutated, 17 are changed to charged amino acids and 15 to polar sidechains (Table 3). In the second group, a polar or charged amino acid is typically replaced by a hydrophobic side chain (but in some cases a different polar/charged sidechain) and modelling suggests that these mutations often remove hydrogen bonds or salt bridges that stabilise the protein structure (Fig. 4f-i). Of the 45 polar or charged residues that are mutated, 33 are likely to result in loss of hydrogen bonding (Additional file 1: Table S8) and half of them (23) are changed to hydrophobic amino acids (Table 3). Examples of these mutations include p.Thr189Met, p.Thr216Met, p.Thr341Ala, p.Arg365Leu, p.Arg452Trp, p.Ser455Leu, p.Ser547Leu (examples shown in Fig. 4f-i; Additional file 1: Table S8).

**Table 3:** Type of amino acid change for mutations in rBAT

	Mutation Amino Acid Type					
Original Amino Acid Type		Hydrophobic	Polar	Positive	Negative	Total
	Hydrophobic	17	15	13	4	49
	Polar	10	2	6	2	20
	Positive	10	5	2	0	17
	Negative	3	1	4	0	8

The remaining mutations either change the polarity or charge of the sidechain (Table 3) or result in a considerable change in the size of the sidechain. Of the 94 mutations only 21 remain in the same group (i.e. hydrophobic, polar, positive or negative charge; Table 3). For the 17 mutations that replace a hydrophobic amino acid with another hydrophobic sidechain, five see a considerable increase in sidechain size (e.g., p.Leu256Phe, p.Gly645Ala) and for a further six the size of the sidechain is reduced (e.g. p.Tyr124Cys, p.Trp255Cys (Fig. 4i), p.Tyr480Cys).

The initial sequence analysis suggested clustering of mutations (Fig. 1). This was also apparent from the structural analysis. There are multiple examples of mutated residues that are close in three dimensions that are not adjacent in sequence. These include: p.Tyr124-p.Tyr151-p.Tyr480, which appear to have Pi interactions between their aromatic sidechains; p.Trp161-p.Asp210, p.Asp179-p.Arg181, p.Arg452-p.Tyr480, p.Arg584-p.Thr417 and p.Tye552His-p.Glu482Lys each of which form a hydrogen bond between them (e.g. Fig. 4g); and p.Phe22-p.Glu268-p.Arg270-p.Arg227 and a large group including residues p.Met467-p.Thr471-p.Leu564-p.Leu567-p.Gly568-p.Tyr582. This clustering suggests that these are regions that either have important structural or functional roles, where mutation of any of them results in changes to protein function.

Given the presence of multiple variants that appear to affect protein stability, mCSM [33] was used to predict the effects of nsSNVs on protein stability. For mCSM, negative ΔΔG values are destabilising for a protein structure, and positive ΔΔG values are stabilising (Fig. 2e-h). rBAT variants known to be associated with cystinuria appear to be distributed more towards negative ΔΔG values than variants present only in ExAC, with median values of −1.122 and −0.668 respectively (*p* = 7.896e-06, Wilcoxon rank sum test) (Fig. 2e & f). Compared to cystinuria associated nsSNVs in b(0+)AT, a greater proportion in rBAT are predicted to be highly destabilizing to the protein (median rBAT value of −1.122, and −0.889 for b(0+)AT) (Fig. 2e & g), supporting the observation that rBAT variants are more likely to destabilize the protein structure. However, this just falls short of statistical significance (*p* = 0.05068, Wilcoxon rank sum test).

### Automated prediction of effects of mutations in rBAT and b(0+)AT

There are many automated methods available to predict the effect of non-synonymous SNVs. Six of these methods, SIFT, PolyPhen-2, MutationAssessor, FATHMM, Condel, and CADD (note Condel and CADD are consensus methods that combine the output from multiple individual predicton methods to generate an overall prediction) were used to predict the effect of each of the mutations present in rBAT and b(0+)AT (see methods). This was done to compare the predictions made with clinical data from a cohort of 74 patients in a UK cystinuria clinic [28]. The predictions made by each of the methods are summarised in Table 4 (full predictions in Additional file 1: Tables S9 and S10).

**Table 4:** Summary of effects of mutations predicted by the automated methods

	rBAT	b(0+)AT
SIFT – Tolerated	23	11
SIFT – Damaging	70	48
PolyPhen2 (HumVar) – benign	12	14
PolyPhen2 (HumVar) – possibly damaging	15	16
PolyPhen2 (HumVar) – probably damaging	66	29
PolyPhen2 (HumDiv) – benign	8	15
PolyPhen2 (HumDiv) – possibly damaging	11	4
PolyPhen2 (HumDiv) – probably damaging	74	40
MutationAssessor – neutral	2	4
MutationAssessor – low	17	6
MutationAssessor – medium	40	29
MutationAssessor - high	34	20
FATHMM – Neutral	0	0
FATHMM – Damaging	93	59
Condel - Neutral	1	7
Condel – Damaging	92	52
CADD- Neutral	5	6
CADD - Deleterious	88	53

For 20 b(0+)AT (of 58) and 31 (of 94) rBAT mutations all methods make the most deleterious predictions. No mutations in either protein were predicted by all six methods to have the lowest or mildest effect on function. For both proteins, the methods agree for a similar proportion of mutations (32.9% for b(0+)AT, 34.5% for rBAT).

The effects of three mutations in b(0+)AT (p.Gly105Arg, p.Ala182Thr and p.Arg333Trp) have been experimentally characterised. We compared the predictions with the known effects (Table 5) and observed good agreement. For b(0+)AT two (p.Gly105Arg and p.Arg333Trp) of the three characterised mutations reduce amino acid transport to 10% of wild type and for both of these mutations all methods predict the greatest effect (Table 5). The third mutation (p.Ala182Thr) reduces transport to 60% of wild type and three methods predict that this mutation will have a limited or no effect on the protein, and three predict that it will be damaging.

**Table 5:** – Comparison of predictions with known experimentally characterised effect

Mutation	Known effect om amino acids transport	SIFT	PolyPhen-2	Mutation Assessor	FATHMM	Condel	CADD
b(0+)AT – G105R	reduced to 10% of WT	Damaging	Probably Damaging	Medium	Damaging	Damaging	Damaging
b(0+)AT A182T	reduced to 60% of WT	Tolerated	Benign	Low	Damaging	Damaging	Damaging
b(0+)AT R333W	reduced to 10% of WT	Damaging	Probably Damaging	High	Damaging	Damaging	Damaging

### Comparison of functional effect predictions with patient phenotype

We considered how well the predicted effects of the mutations agreed with the observed phenotypes of patients, based on the grouping of the patients by the prediction scores (see methods). Each mutation was assigned a severity score of either 1 (mild) or 2 (severe) based on the prediction score from the predictive method. Then for each individual an overall severity score was calculated based on the mutations present (see methods). The patients were grouped according to their overall score and the phenotype data compared between the different groups (see methods).

As the predictive methods associate a score (or probability) with their predictions we first investigated how altering the threshold between assigning a mutation to the mild or severe group affected the outcome of the comparisons. For each mutation effect prediction method, the number of patients in each severity score group stabilised over a range of prediction score thresholds, e.g. for PolyPhen2 the groupings are stable between thresholds of ≥0.65 and ≥0.80. A single threshold within these ranges was then chosen as the cutoff between mild and severe mutations for that method and used for comparison (Table 1 and Additional file 1: Tables S3 and S4).

**Fig. 5:**
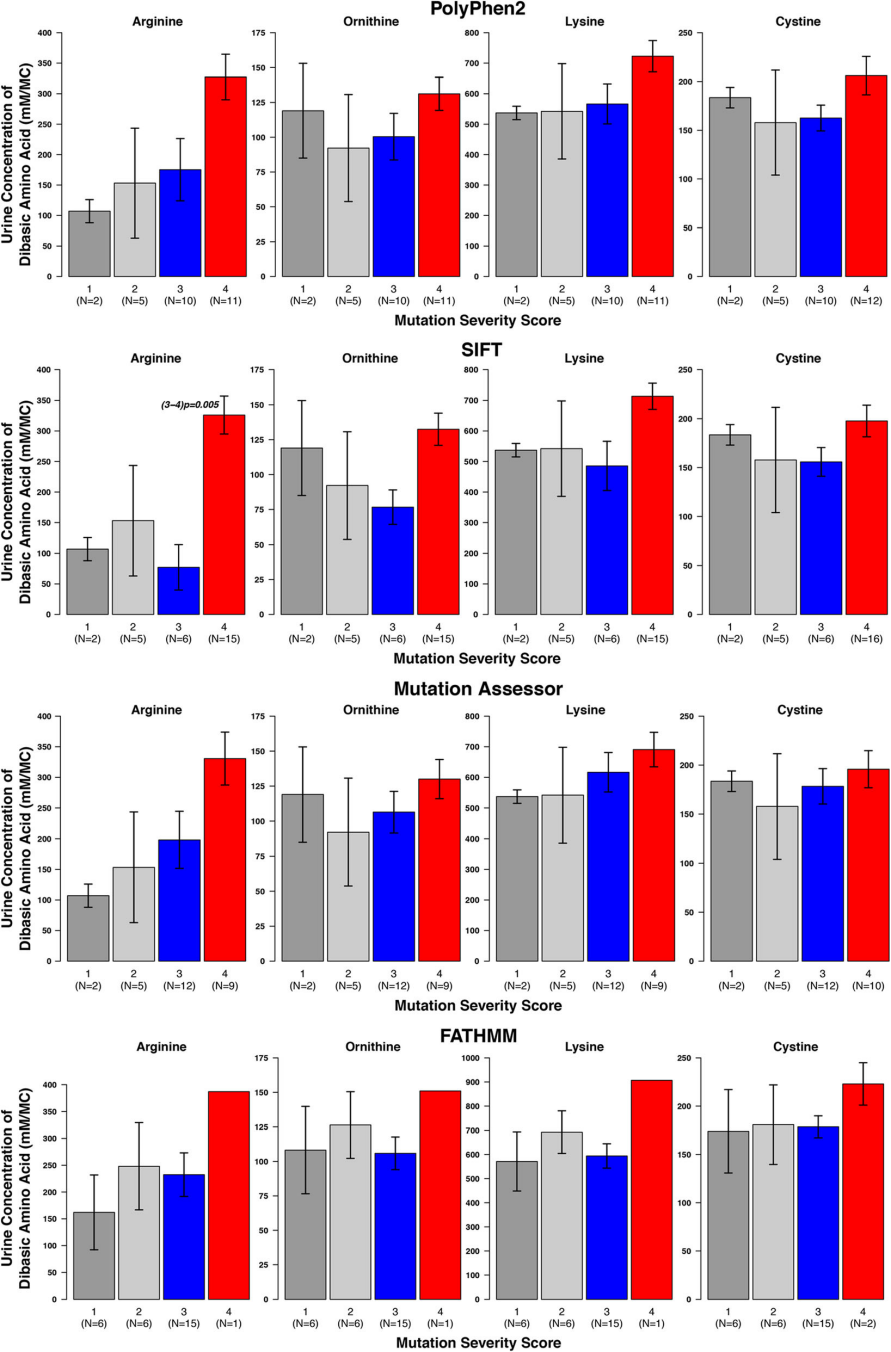
Comparison of average urine levels of amino acids between the different severity score groups, for individuals with b(o+)AT mutations. There is one plot per prediction method (PolyPhen2, SIFT Mutation Assessor, and FATHMM). The group numbers are given at the bottom of the plots, with the sample number given in brackets underneath the group name. Where significant differences between groups occur (*p* < 0.05) the *p*-value is displayed on the plot, e.g. (1–2)*p* = 0.001 means a significant difference between groups 1 and 2

**Fig. 6:**
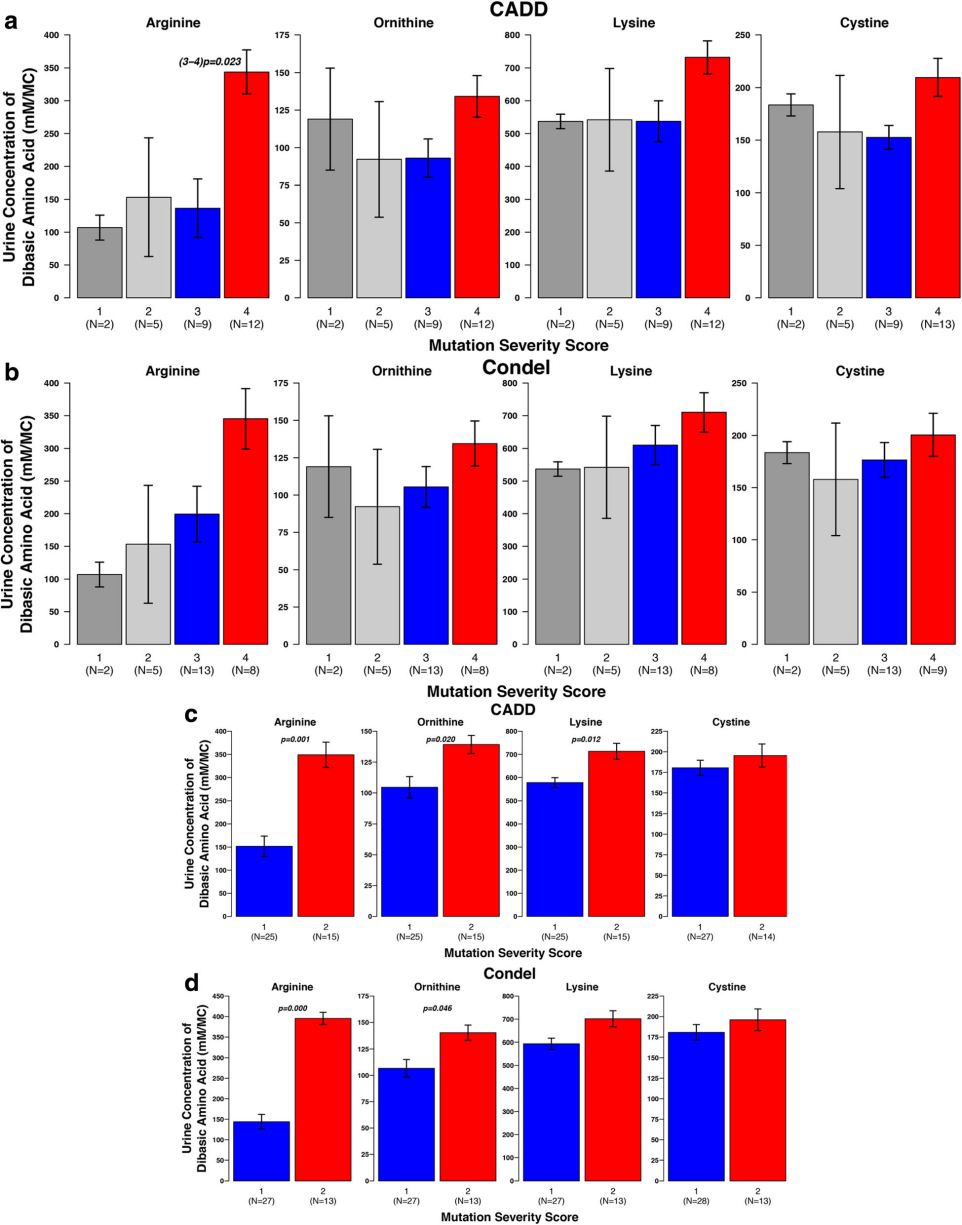
Comparison of average urine levels of amino acids between the different severity score groups using Condel and CADD. **a** and **b** Individuals with b(o+)AT mutations. **c** and **d** Individuals with rBAT mutations. There is one plot per integrated prediction method (CADD and Condel) for each gene. The group numbers are given at the bottom of the plots, with the sample number given in brackets underneath the group name. Where significant differences between groups occur (*p* < 0.05) the *p*-value is displayed on the plot, e.g. (1–2) *p* = 0.001 means a significant difference between groups 1 and 2

First b(0+)AT was considered, (Figs. 5, 6a, and Additional file 2: Figs. S2, and S3). Each of the prediction methods sorted the patients in to slightly different groupings, but various general trends were observed across the methods. Considering the urinary levels of the amino acids arginine, ornithine, lysine and cystine, for all methods there was a general trend for the levels to be higher in the high severity score groups. For each method the difference seems greatest for arginine, and for SIFT and CADD there was a significant difference between severity score groups 3 and 4 for the levels of arginine (Figs. 5 and 6a).

The age of diagnosis and the number of stone episodes and interventions over a three-year period were also considered. Again, the average values for the higher severity score groups typically followed the pattern that may be expected i.e. lower severity score group have a later age of presentation and lower number of stone episodes and interventions (Additional file 2: Figs. S2 & S3). Many of these values have large ranges (as demonstrated by the error bars), but some of these comparisons showed statistical significance, e.g. PolyPhen2 predicted groups 3 and 4 show a significant difference for the number of interventions (*p* = 0.017) and age of diagnosis for Mutation Assessor and Condel predicted groups 3 and 4 (*p* = 0.015 and *p* = 0.014, respectively).

**Fig. 7:**
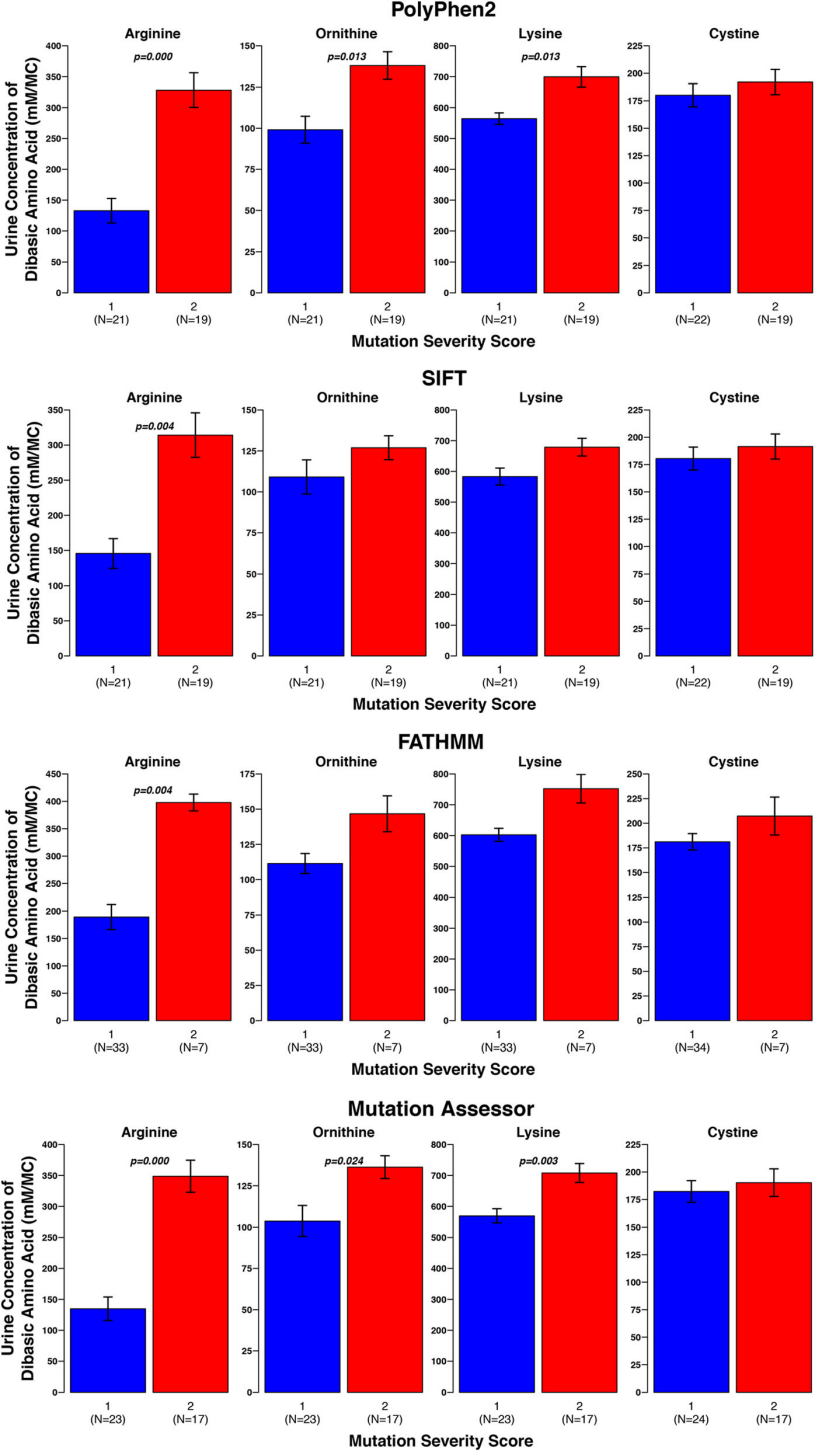
Comparison of average urine levels of amino acids between the different severity score groups, for individuals with rBAT mutations. There is one plot per prediction method (PolyPhen2, SIFT Mutation Assessor, and FATHMM). The group numbers are given at the bottom of the plots, with the sample number given in brackets underneath the group name. Where significant differences between groups occur (*p* < 0.05) the *p*-value is displayed on the plot, e.g. (1–2)*p* = 0.001 means a significant difference between groups 1 and 2

The equivalent analysis was performed for patients with mutations in rBAT. For rBAT there are only two categories – mild (score 1) and severe (score 2) (details in methods). For all of the urinary amino acid levels and for all predictive methods the average for severity score group 1 was lower than for severity score group 2 (Figs. 6b & 7). These differences in arginine levels were statistically significant across all methods. The differences in ornithine and lysine levels were statistically significant for PolyPhen2, Mutation Assessor, and CADD, and the difference in ornithine was significant for Condel. No method found a statistically significant difference between the groups for cystine levels. However, there are difficulties in the accurate measurement of cystine, so this result is unlikely to be reliable and the other levels of other amino acids are a better indicator of disease severity.

Patients in severity score group 1 for all methods present with disease at a later age than those in severtity group 2, though these differences are not statistically significant (Additional file 2: Figs. S4 & S5). There is little difference between the average number of stone episodes for the two groups (for all predictive methods) and for SIFT the number of interventions is greater in the lower severity score group (Additional file 2: Figs. S4 & S5).

All methods struggle to identify differences in the age of diagnosis, number of stone episodes and number of interventions, but they are better at finding differences in the urinary amino acid levels between the severity score groups. This is perhaps because urinary amino acid levels are a less complex phenotype, which was directly measured. In contrast the other phenotypes are less easily measured or recorded. For example, a patient may present with a large number of stones which all pass spontaneously, whereas another patient may present with fewer more serious stone episodes. The measurements taken may depend on patients accurately recording the number of stones that pass and medical interventions may also affect the number of stone episodes. Therefore, comparisons of urinary amino acid levels may be more informative of disease severity.

## Discussion

We have surveyed the mutations present in SLC7A9 and SLC3A1 and their likely effect on the encoded proteins b(0+)AT and rBAT. Across 49 studies, 58 and 94 cystinuria associated point mutations were identified in SLC7A9 and SLC3A1, respectively. Our initial comparison of cystinuria associated variants with variants present only in ExAC, showed that the disease associated variants typically have a lower frequency in the population, they tend to cluster in the protein sequence, largely in different areas of the protein sequence to the ExAC variants which show less clustering. This may suggest that particular regions of the protein sequence cannot be altered without affecting protein function. Additionally, we found that the frequency of ExAC variants was higher for SLC3A1, which may represent the autosomal recessive inheritance of cystinuria when caused by SLC3A1 mutations.

Using structural models to investigate mutations in rBAT was more complicated than for b(0+)AT because the function of rBAT is not clearly understood. Interestingly in rBAT, few mutations directly affected the predicted functional residues that would be associated with the enzyme activity that is typically present in this family of proteins. However, a large number of mutations either remove hydrogen bonds or introduce a charge into a buried or hydrophobic region and could therefore disrupt protein folding or reduce stability. This is consistent with what we know of the rBAT protein; experimental studies suggest that the heavy rBAT subunit is essential for cell surface expression of b(0+)AT and essential for transport of the heterodimer to the plasma membrane [3]. The extracellular glycosidase domain may only have a role in cystine transport [5] and the requirement of chaperone for rBAT to fold correctly. A number of rBAT mutations have been linked with incorrect folding of the protein and/or trafficking to the plasma membrane [6].

In contrast, it is known that the light chain b(0+)AT encoded by SLC7A9 forms the exchanger of dibasic amino acids for neutral amino acids [47]. In fact, it has been suggested that the light subunit may be fully functional even in the absence of the heavy subunit [8, 9, 48, 49]. Given the important functional role of b(0+)AT in amino acid transport, multiple cystinuria associated mutations are identified that affect or are close to predicted functional residues. Additionally, other mutations either seem likely to result in conformational changes or affect protein stability. For example, there are many examples where a buried hydrophobic amino acid is replaced by a charged or polar one but in contrast to rBAT there are few mutations that remove hydrogen bonding, which is likely because b(0+)AT is highly hydrophobic.

Comparison between the different variant effect predictors indicated that they agree for approximately 30–35% of point mutations. The investigation of using these methods to classify patients’ disease into mild and severe, has a number of limitations. The methods used have been developed to predict amino acid changes that are likely to cause disease and we see that they do this fairly well for the mutations considered, with most of them predicted to be deleterious. So, we have not used them here for exactly the role they were developed. The scoring system may be overly simple, a patient with two low severity mutations will perhaps fair better than an individual with one severe mutation (or vice versa), but they are treated equally in our scoring system. Additionally, the sample size is relatively small. Finally, the complex inheritance patterns of b(0+)AT makes predictions of mutation effect harder. For rBAT, the pattern is clearer with high severity score groups tending to worse phenotypes (see Figs. 6b, 7, and Additional file 2: Figs. S4, and S5).

However, given these limitations the analysis suggests the potential for the use of such methods in this way. Typically, we observed the phenotype differences that would be expected, if those predicted in the lower severity score group actually had a milder form of the disease. For example, the urine levels of nearly all of the amino acids considered (for most of the methods) across both proteins, are lower for the lower severity score group (but not all are statistically significant). Additionally, for rBAT and b(0+)AT there are general trends for most methods where the age of presentation is higher in the low severity score groups, while the number of stone episodes and number of interventions is greater in the high severity score groups, but this is mostly not statistically significant, which highlights the need to use a larger cohort.

## Conclusion

Overall these results are promising. Methods used to predict if mutations are deleterious have been used to categorise mutations and there is some correlation with phenotype. However, it also highlights the limitations of existing methods and improvements are required if they are to be used for even relatively simple precision medicine applications such as the classification of cystinuria disease severity. Additionally, the analyses performed here need to be expanded into a larger cohort of individuals to obtain greater confidence and to identify the most effective way to categorise individuals. With a larger dataset there would be the potential to train a method specifically to classify individuals based on their SLC3A1 or SLC7A9 mutations, which may be more effective than trying to use existing methods that have not been designed specifically to do this. Following this there is the potential to investigate the use of such an approach to provide individual precision treatment in the clinic.

## Additional files

Additional file 1: All supplementary tables for the manuscript. (XLSX 427 kb)

Additional file 2: All supplementary figures for the manuscript. (PDF 640 kb)
