# Supplementary material for: Associating mutations causing cystinuria with disease severity with the aim of providing precision medicine
Source: BMC Genomics. 2017 Aug 11;18(Suppl 5):550. doi: 10.1186/s12864-017-3913-1 (PMC5558187; doi:10.1186/s12864-017-3913-1)
Supplement: Supplementary file 2 — All supplementary figures for the manuscript. (PDF 640 kb) [file 12864_2017_3913_MOESM2_ESM.pdf]

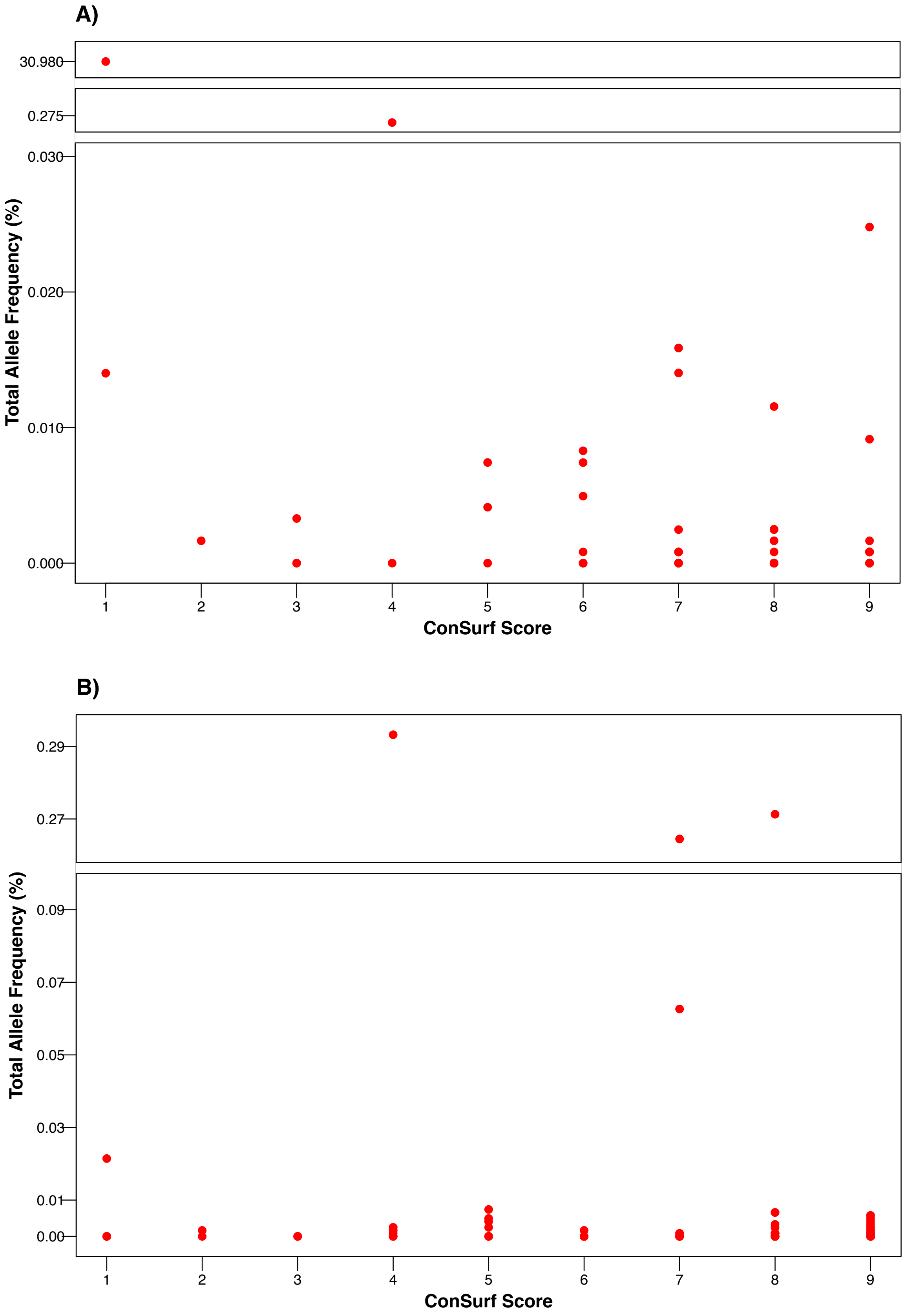

Figure S1. Comparison of allele frequency (based on ExAC) and evolutionary conservation (based on ConSurf scores). A) Cystinuria associated mutations of b(0+)AT. B) Cystinuria associated mutations of rBAT.

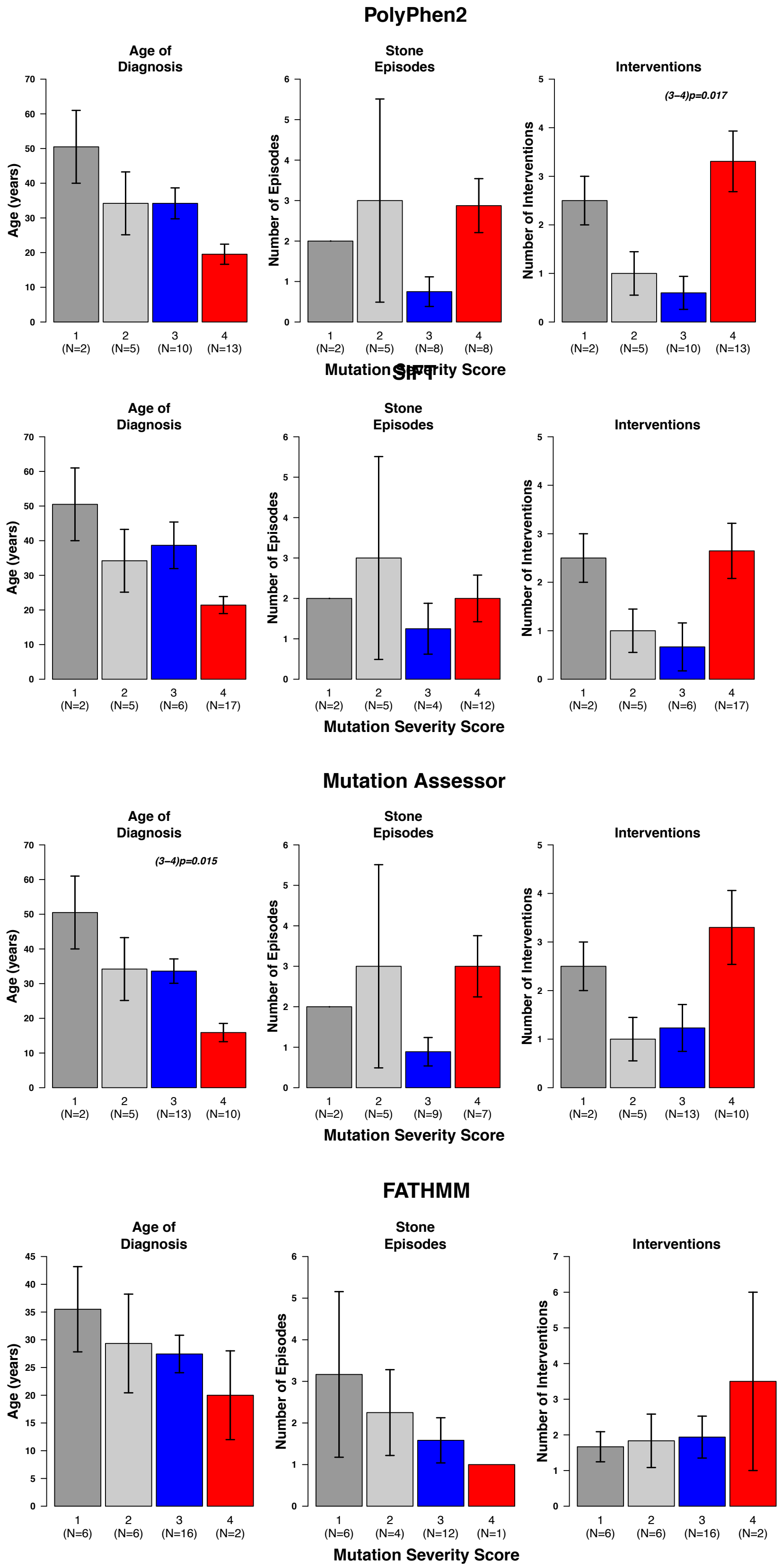

Figure S2. Comparison of other clinical parameters between the different severity score groups, for individuals with b(o+)AT mutations. There is one plot per prediction method (PolyPhen2, SIFT Mutation Assessor, and FATHMM). The group numbers are given at the bottom of the plots, with the sample number given in brackets underneath the group name. Where significant differences between groups occur ($p<0.05$) the p-value is displayed on the plot, e.g. (1-2)p=0.001 means a significant difference between groups 1 and 2.

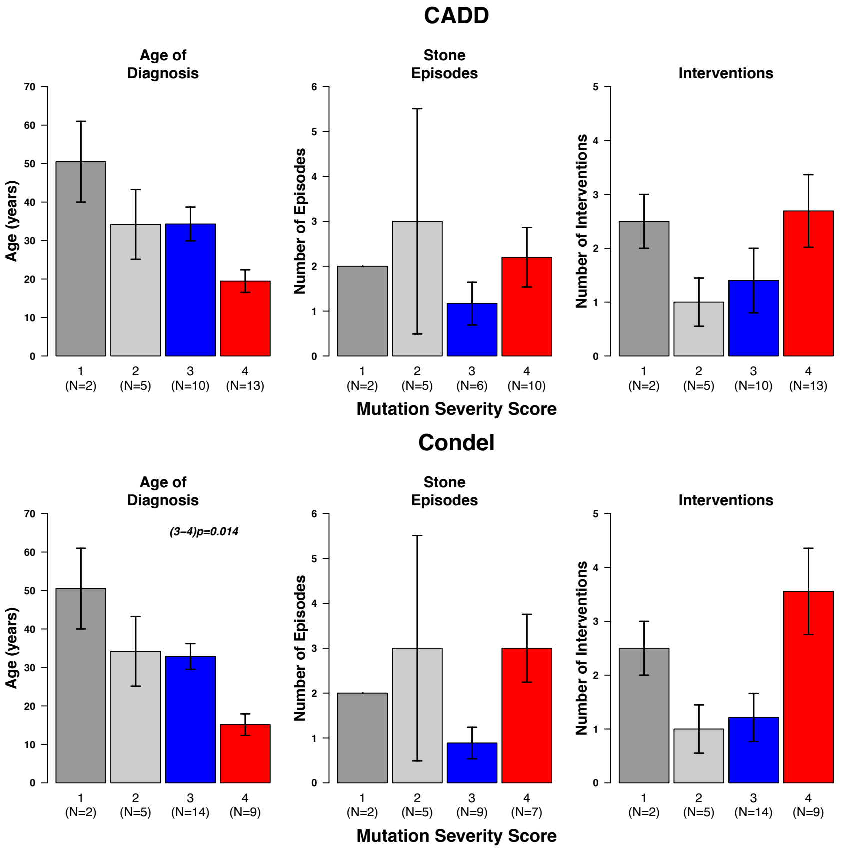

Figure S3. Comparison of other clinical parameters between the different severity score groups, for individuals with b(o+)AT mutations. There is one plot per integrated prediction method (CADD and Condel). The group numbers are given at the bottom of the plots, with the sample number given in brackets underneath the group name. Where significant differences between groups occur ($p<0.05$) the p-value is displayed on the plot, e.g. (1-2)p=0.001 means a significant difference between groups 1 and 2.

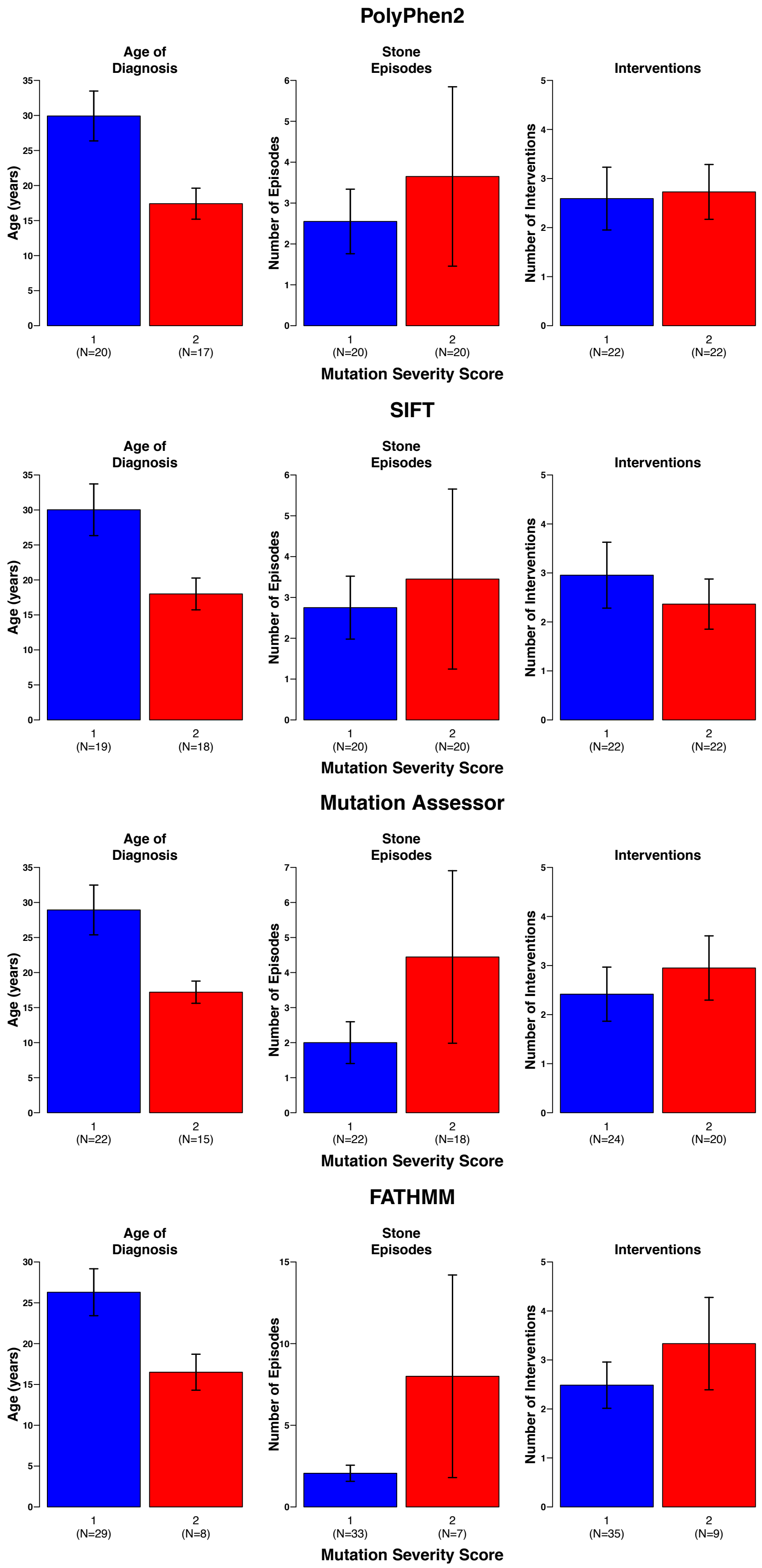

Figure S4. Comparison of other clinical parameters between the different severity score groups, for individuals with rBAT mutations. There is one plot per prediction method (PolyPhen2, SIFT Mutation Assessor, and FATHMM). The group numbers are given at the bottom of the plots, with the sample number given in brackets underneath the group name. Where significant differences between groups occur (p<0.05) the p-value is displayed on the plot, e.g. (1-2)p=0.001 means a significant difference between groups 1 and 2.

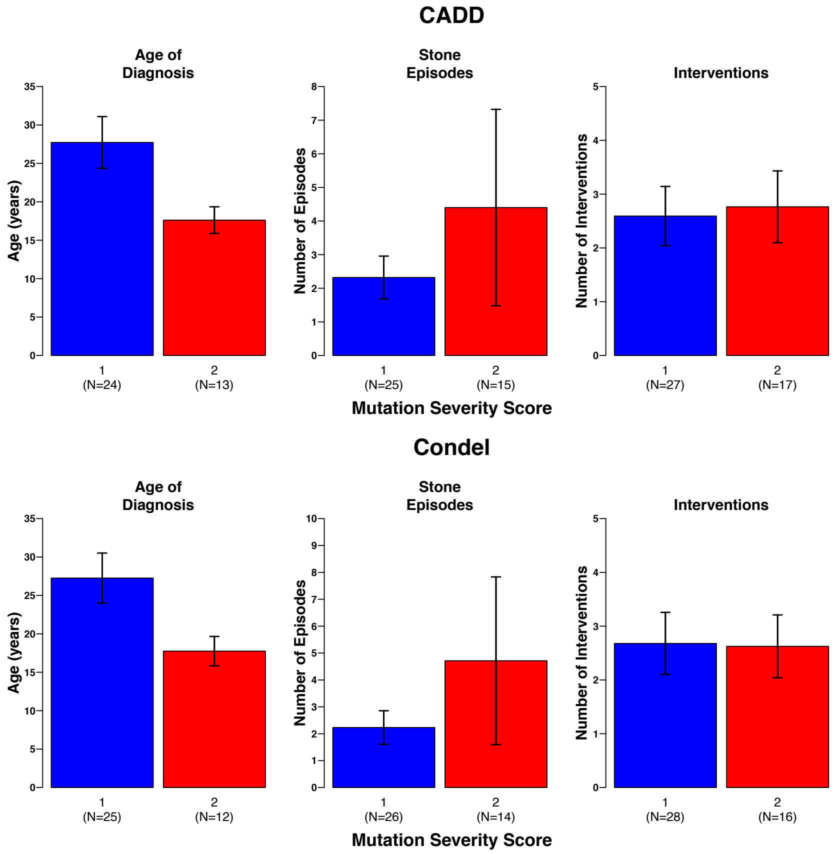

Figure S5. Comparison of other clinical parameters between the different severity score groups, for individuals with rBAT mutations. There is one plot per integrated prediction method (CADD and Condel). The group numbers are given at the bottom of the plots, with the sample number given in brackets underneath the group name. Where significant differences between groups occur ($p<0.05$) the p-value is displayed on the plot, e.g. (1-2)p=0.001 means a significant difference between groups 1 and 2.
